# Supplementary material for: Dispersion-engineered metasurfaces reaching broadband 90% relative diffraction efficiency
Source: Nat Commun. 2023 May 3;14:2544. doi: 10.1038/s41467-023-38185-2 (PMC10156701; doi:10.1038/s41467-023-38185-2)
Supplement: Supplementary file 1 — Supplementary Information [file 41467_2023_38185_MOESM1_ESM.pdf]

Supplementary Information for:

Dispersion-engineered Metasurfaces Reaching Broadband 90% Relative Diffraction Efficiency

Wei Ting Chen[1,+], Joon-Suh Park[1,+], Justin Marchioni[1,2], Sophia Millay[1,3], Kerolos M.A. Yousef[1] and Federico Capasso[1]

[1]*Harvard John A. Paulson School of Engineering and Applied Sciences, Harvard University, Cambridge, Massachusetts 02138, USA*

[2]*University of Waterloo, Waterloo, ON N2L 3G1, Canada*

[3]*Department of Physics, Williams College, Williamstown, Massachusetts 01267, USA*

[+]*Equal contribution*

[*]*Corresponding author: capasso@seas.harvard.edu*

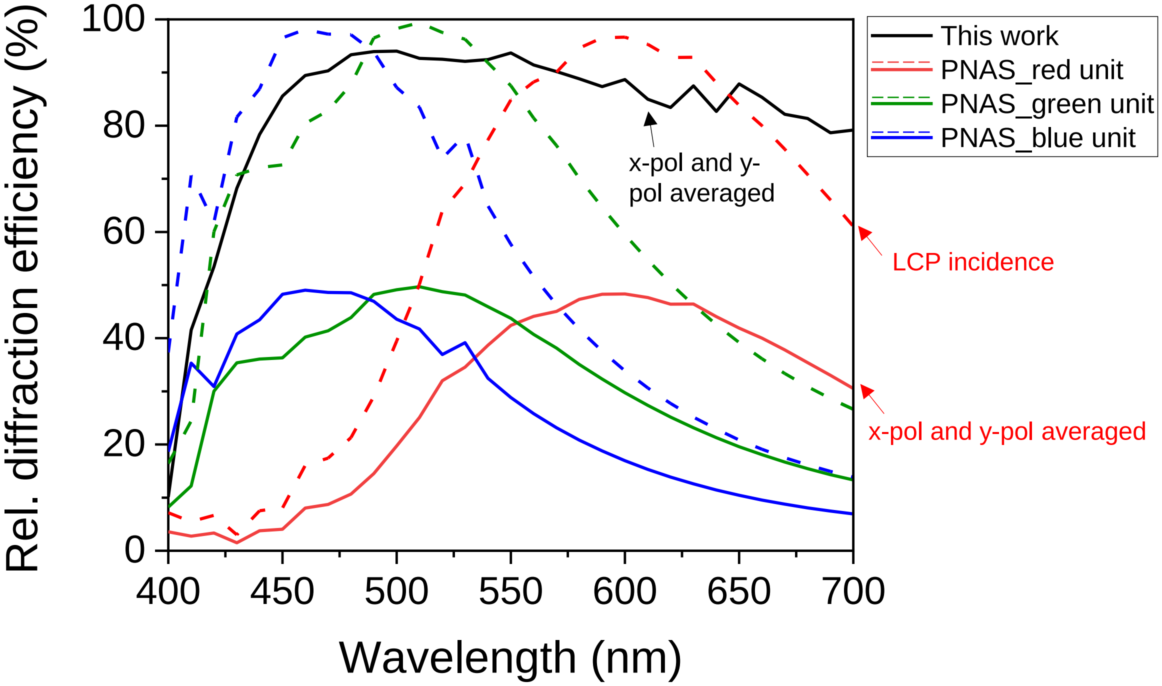

Figure S1: Comparison of simulated relative diffraction efficiency. The black one corresponds to the dispersion-engineered metagrating shown in Fig. 1b. The red, green and blue ones are from three different metagratings reported in Ref. S1 based on three nanofin unit cells, whose (length, width, unit cell size) are (410, 85, 430), (250, 85, 325), (200, 90, 300) in nanometers. The nanofin metagratings are efficient only in left-handed polarized incidence (see the dashed curves). All solid curves are averaged efficiency under x- and y-polarized incident light, while the dashed ones are under left-handed circularly polarized incidence. In comparison with the nanofin metagratings, the dispersion-engineered metagrating is insensitive to incident polarization and has wide bandwidth. After publication, the layouts and Lumerical simulation files can be accessed on the authors' websites at https://www.weitingchen-meta.com/ and https://capasso.seas.harvard.edu/.

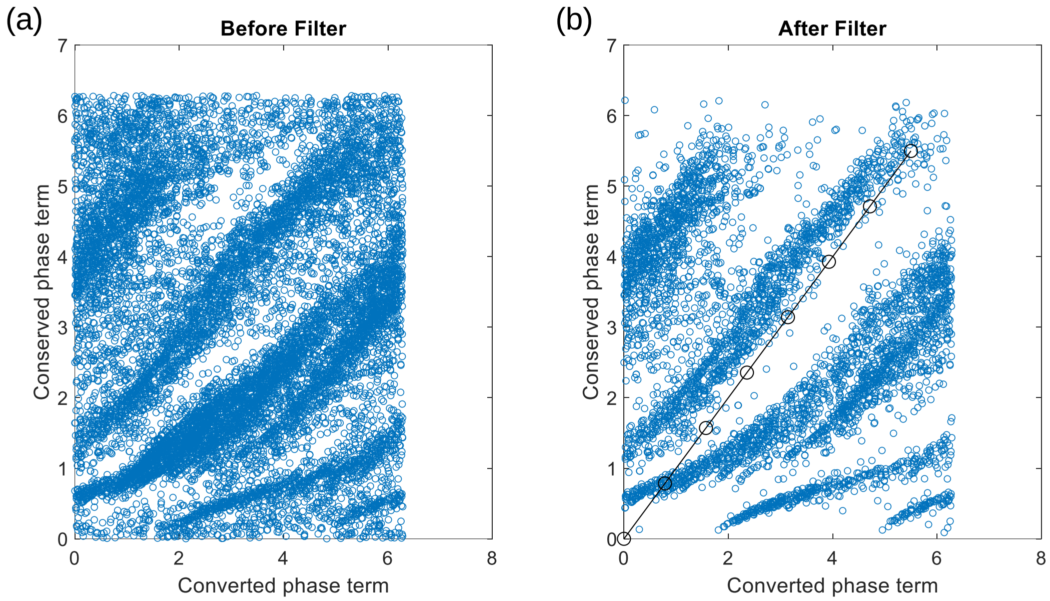

Figure S2: Nanostructure library visualization to elaborate design principle. (a) At design wavelength, each nanostructure (blue circles) is shown by its phases of polarization converted and conserved terms at design wavelengths. It is important to make sure that the library can cover the entire 2π by 2π area. (b) A similar plot after removing the nanostructures with low goodness of fit given by their dispersion properties. This step is to filter those nanostructures with abrupt phase jumps within the design bandwidth. The black circles indicate the target linear phase of grating.

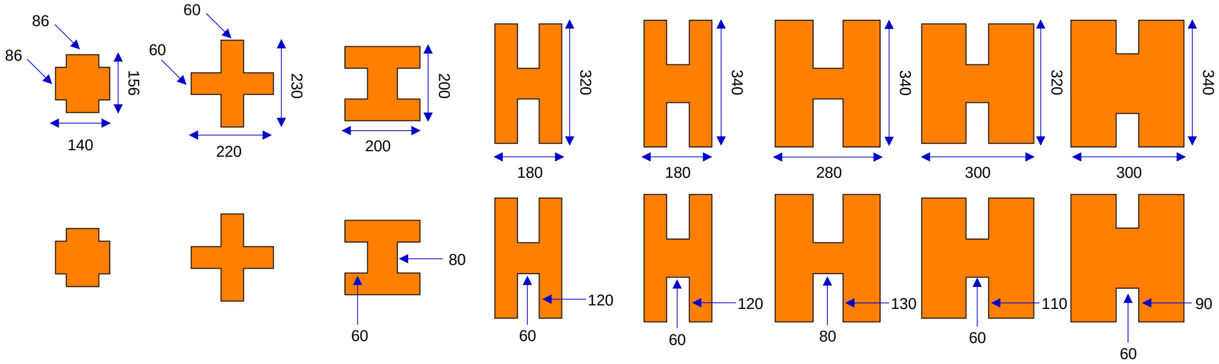

Figure S3: Nanostructure parameters in nanometer unit. After publication, the layout and Lumerical simulation file can be accessed on the authors' websites at https://www.weitingchen-meta.com/ and https://capasso.seas.harvard.edu/. The height of these nanostructures is 600 nm.

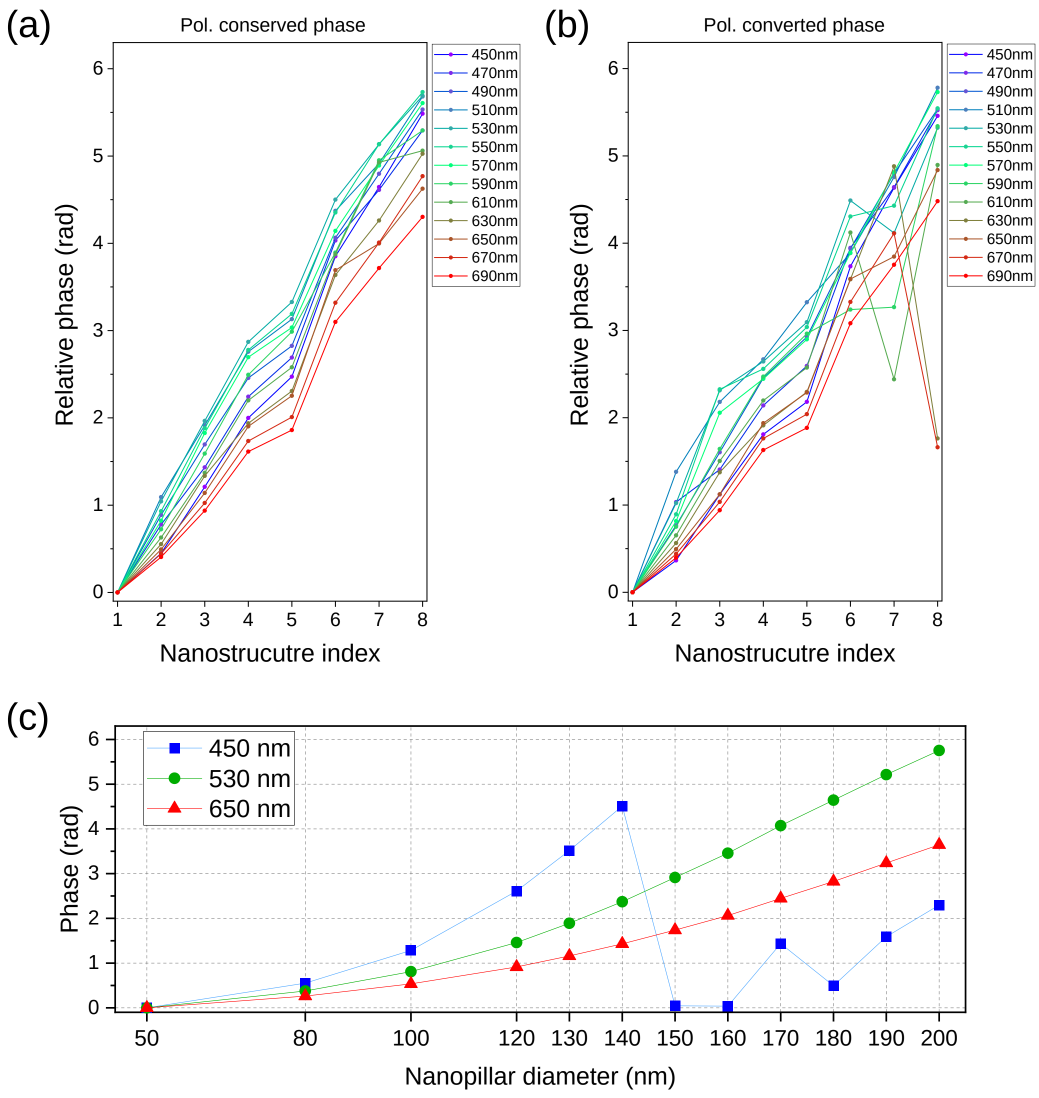

Figure S4: Phase plots of nanostructures. (a) and (b) are the phase of polarization conserved and converted terms, respectively, for each dispersion-engineered nanostructure depicted in Fig. 1b in main text. **(**c) Phases of cylindrical nanopillars showing deviated phase profiles from the design one (green) when incident wavelength moves to red and blue wavelengths (labeled in legend). The metagrating shown in Fig. 1a consists of those nanopillars. Because phase is relative, the phase of the 1st nanostructure is set as zero as a reference.

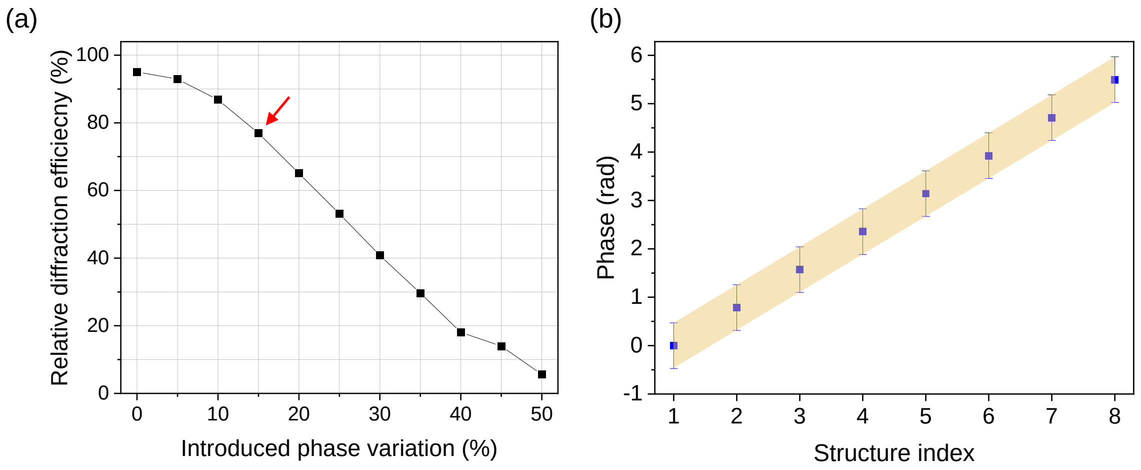

Figure S5: Calculated efficiency as a function of phase errors based on scalar approximation and Fourier transform. (a) Relative diffraction efficiency as a function of introduced phase errors in percentage. The relative diffraction efficiency is shown as a lower bound estimation. For instance, when the value of *x*-axis is 15%, it means a phase error of $\pm 0.15\pi$ is introduced to the ideal linear phase profile resulting in a corresponding relative diffraction efficiency no lower than 77% (red arrow). (b) Phase plot showing the ideal linear phase profile sampled by 8 units (the squares). The size of error bar is $2\times 0.15\pi$ corresponding to the case depicted by the red arrow in (a). In this case, as long as the deviation of the linear phase profile is within the yellow area defined by the error bars, its relative diffraction efficiency is higher than 77%.

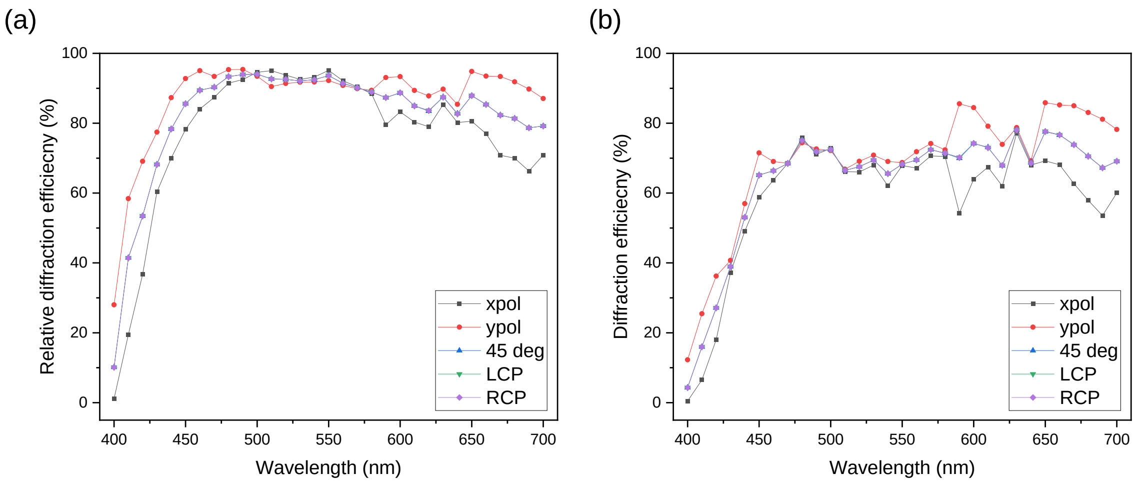

Figure S6: Simulated efficiency plots for the metagrating depicted in Fig. 1b. (a) Relative diffraction efficiency under different incident polarizations in normal incidence. The relative diffraction efficiency is defined as the power diffracted to the 1st order divided by the power of transmitted light. Note that the curves of 45-degree, left-handed and right-handed polarizations are overlapped. (b) Diffraction efficiency under different incident polarizations in normal incidence. The diffraction efficiency is defined as the power diffracted to the 1st order divided by the power of incident light.

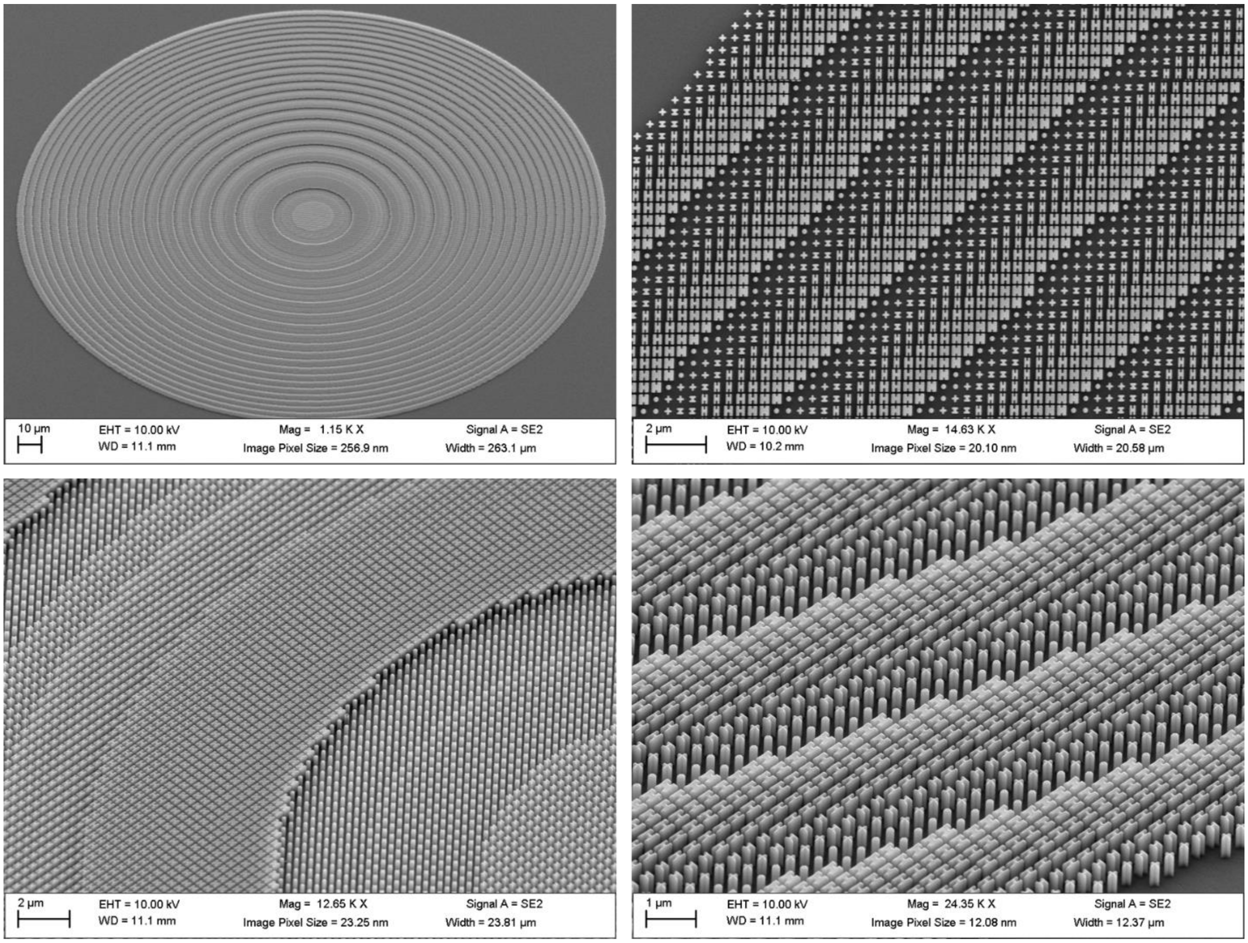

Figure S7: SEM images of a fabricated dispersion-engineered metalenses (NA = 0.15)

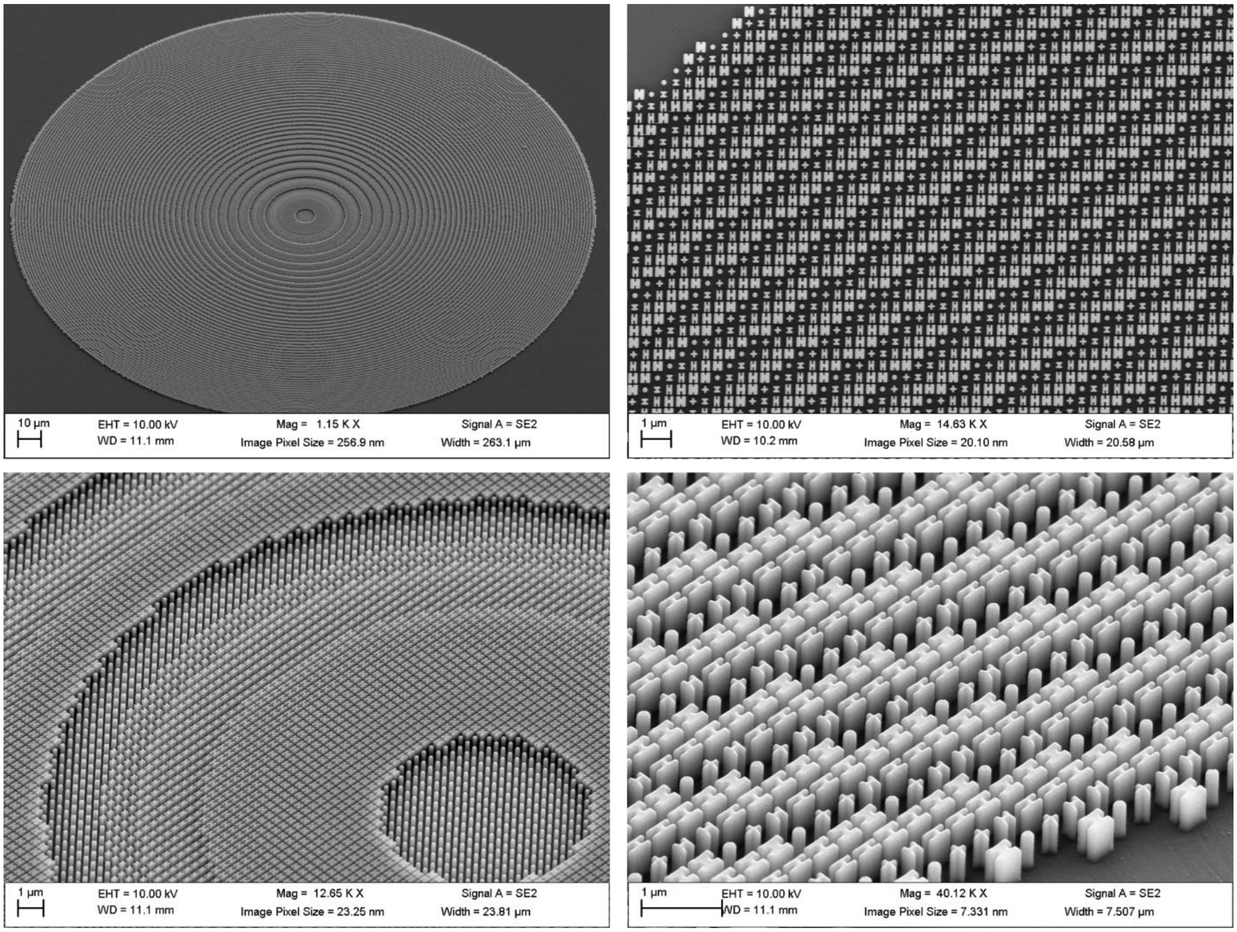

Figure S8: SEM images of fabricated dispersion-engineered metalenses (NA = 0.45)

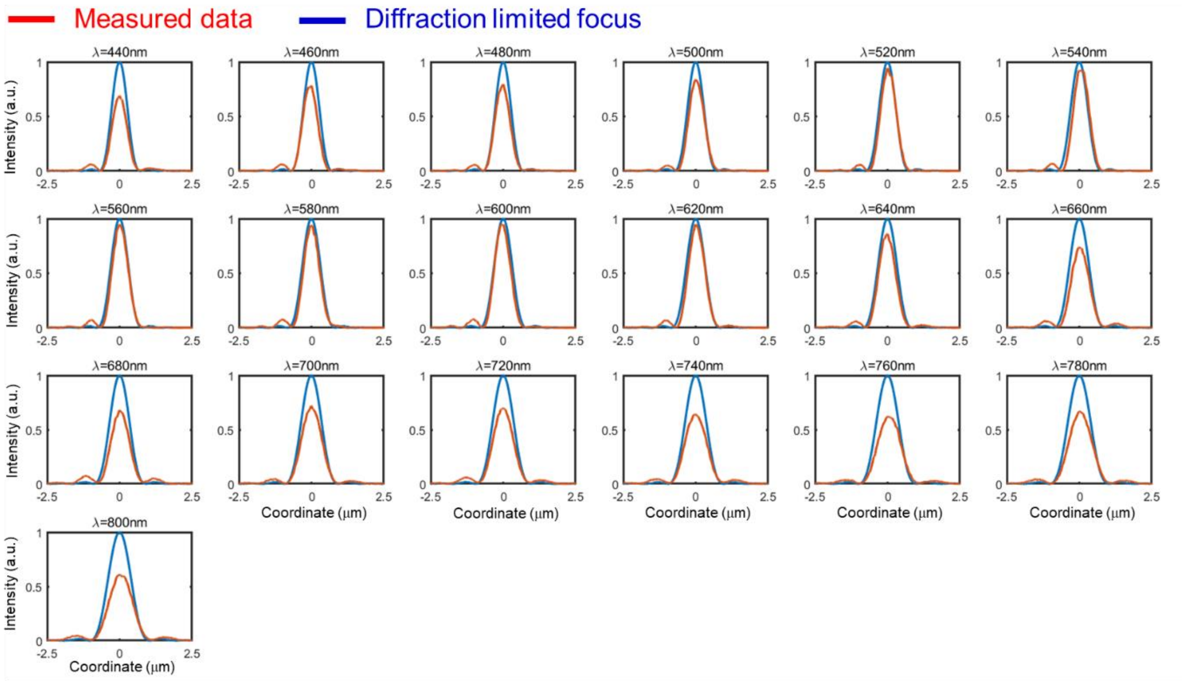

Figure S9: Measured focal spot intensity profile of the NA=0.45 dispersion-engineered metalens shown in Figure 3 (g)-(i).

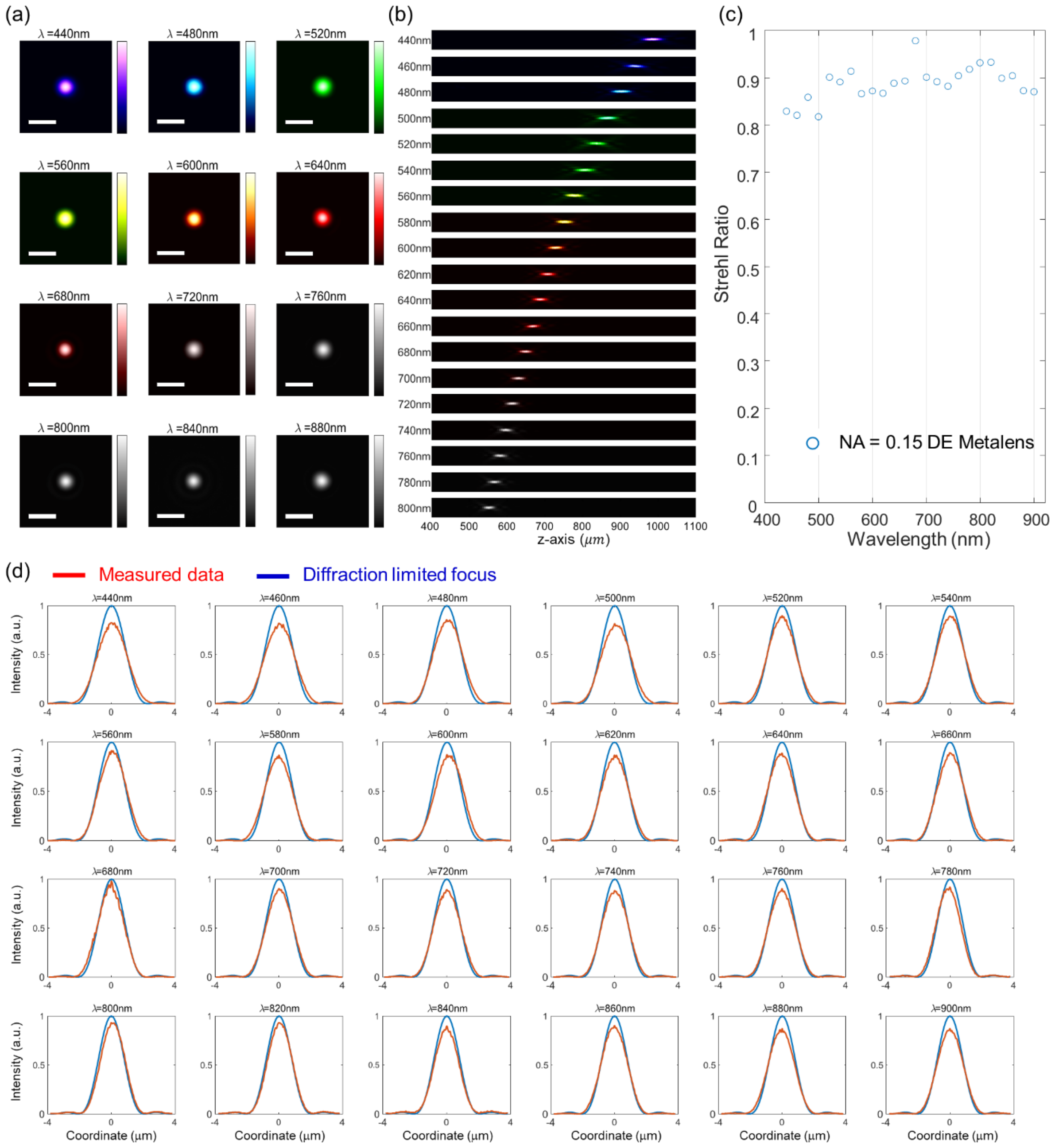

Figure S10: Focal spot images and point-spread-function of dispersion-engineered metalenses (NA=0.15) (a) Measured focal spot images and (b) point-spread-function along the optic-axis of the dispersion-engineered NA=0.15 metalens for different incident wavelengths. Scale bars: 5 μm. (c) Measured Strehl ratios and (d) Measured focal spot intensity profiles (red curves) at different wavelengths (labelled on the top) for the NA = 0.15 dispersion-engineered metalens shown in Fig. 3a in the main text. For diffraction-limited focal spots (the blue curves), their NAs were calculated from measured focal length at each wavelength and the lens diameter of 250 μm.

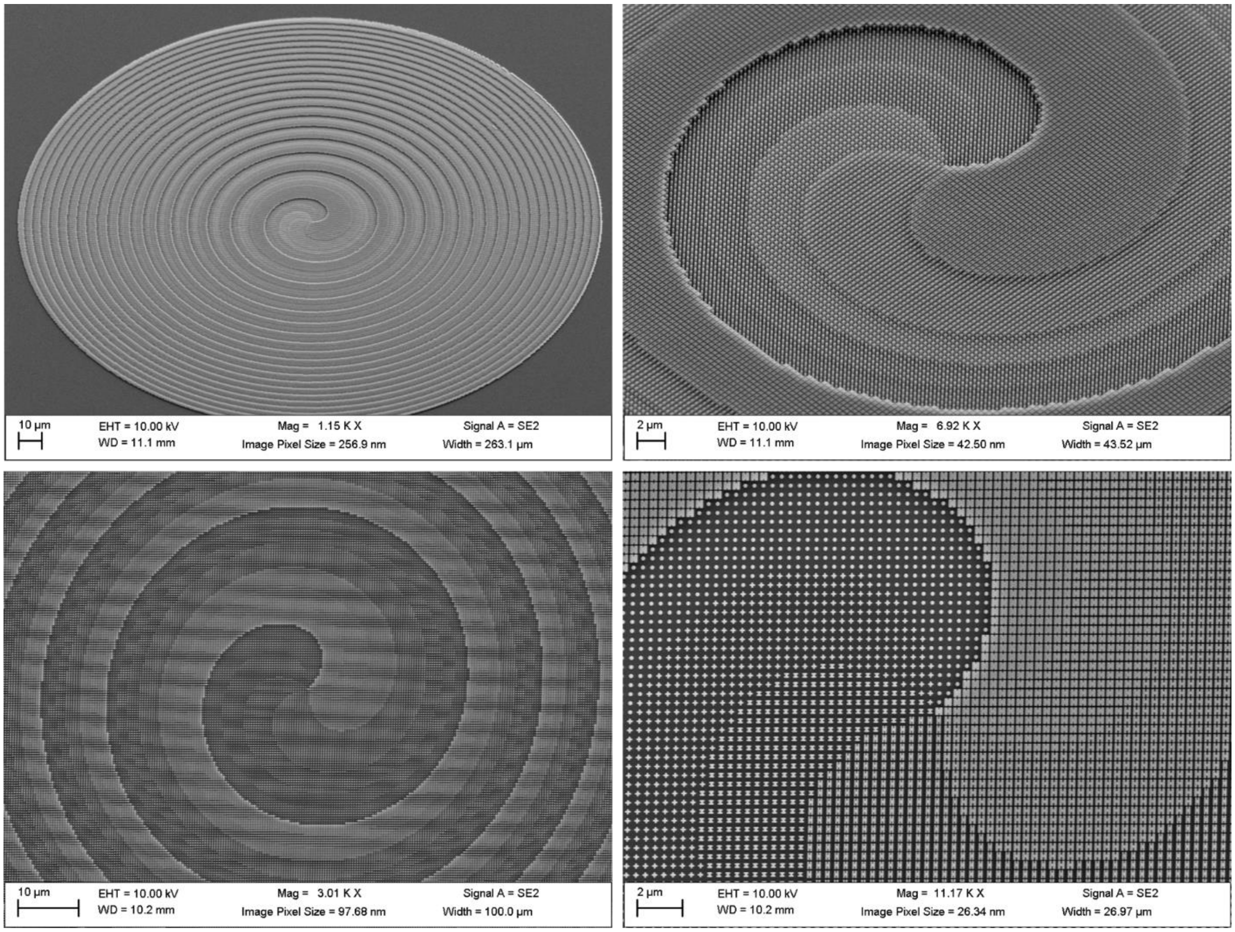

Figure S11: SEM images of fabricated multi-functional, dispersion-engineered metalens, focusing a donut beam having an orbital angular momentum of 1.

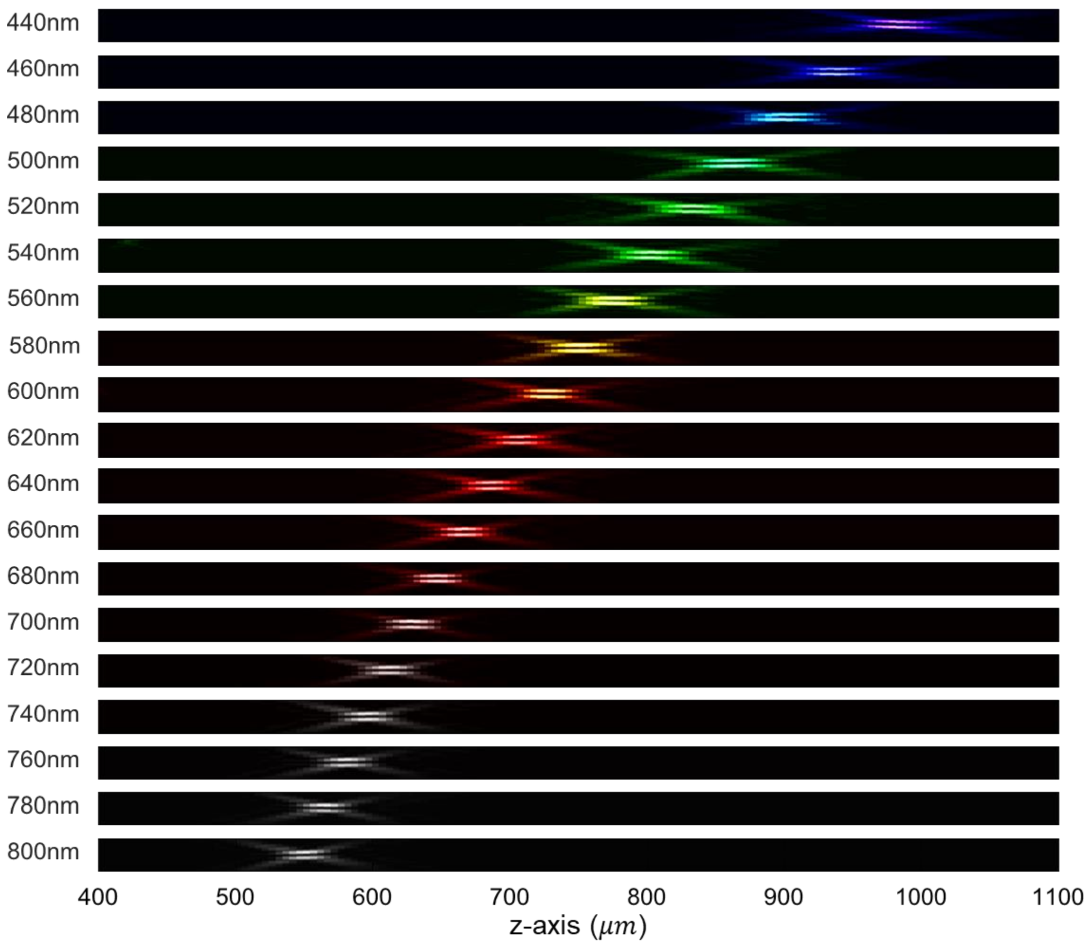

Figure S12. Measured point spread function along the optic-axis for the multifunctional dispersion-engineered metalens, showing a focused donut beam having an orbital angular momentum of 1.

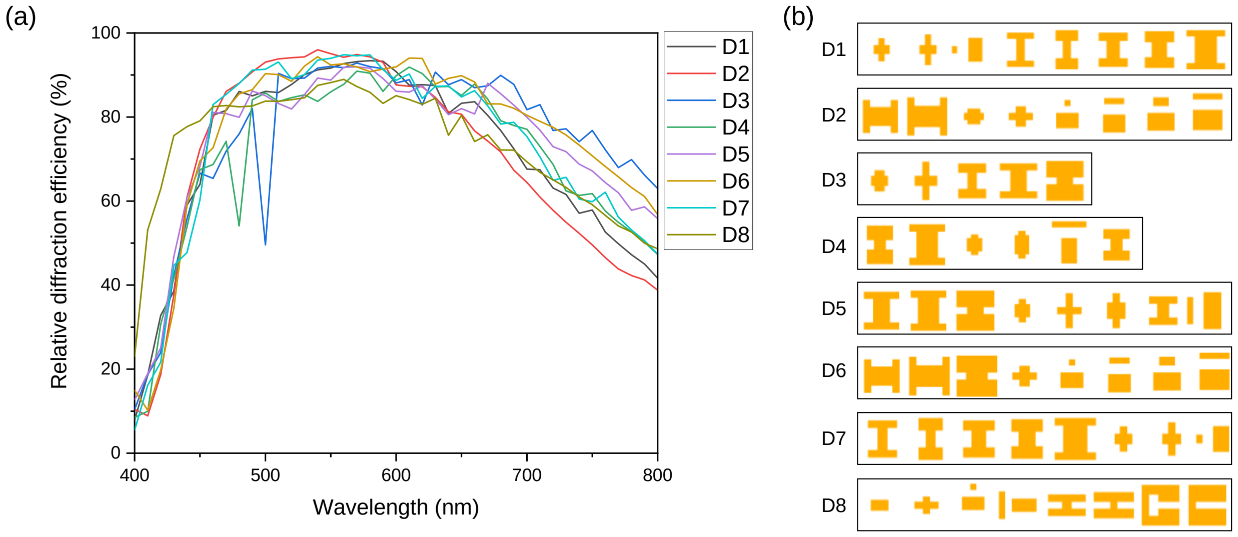

Figure S13: Other dispersion-engineered high-efficiency metagratings. (a) Simulated relative diffraction efficiencies for the metagratings shown in (b). (b) Layouts of the metagratings. After publication, the layouts and Lumerical simulation files can be accessed on the authors' websites at https://www.weitingchen-meta.com/ and https://capasso.seas.harvard.edu/. The height of the nanostructures is 600 nm.

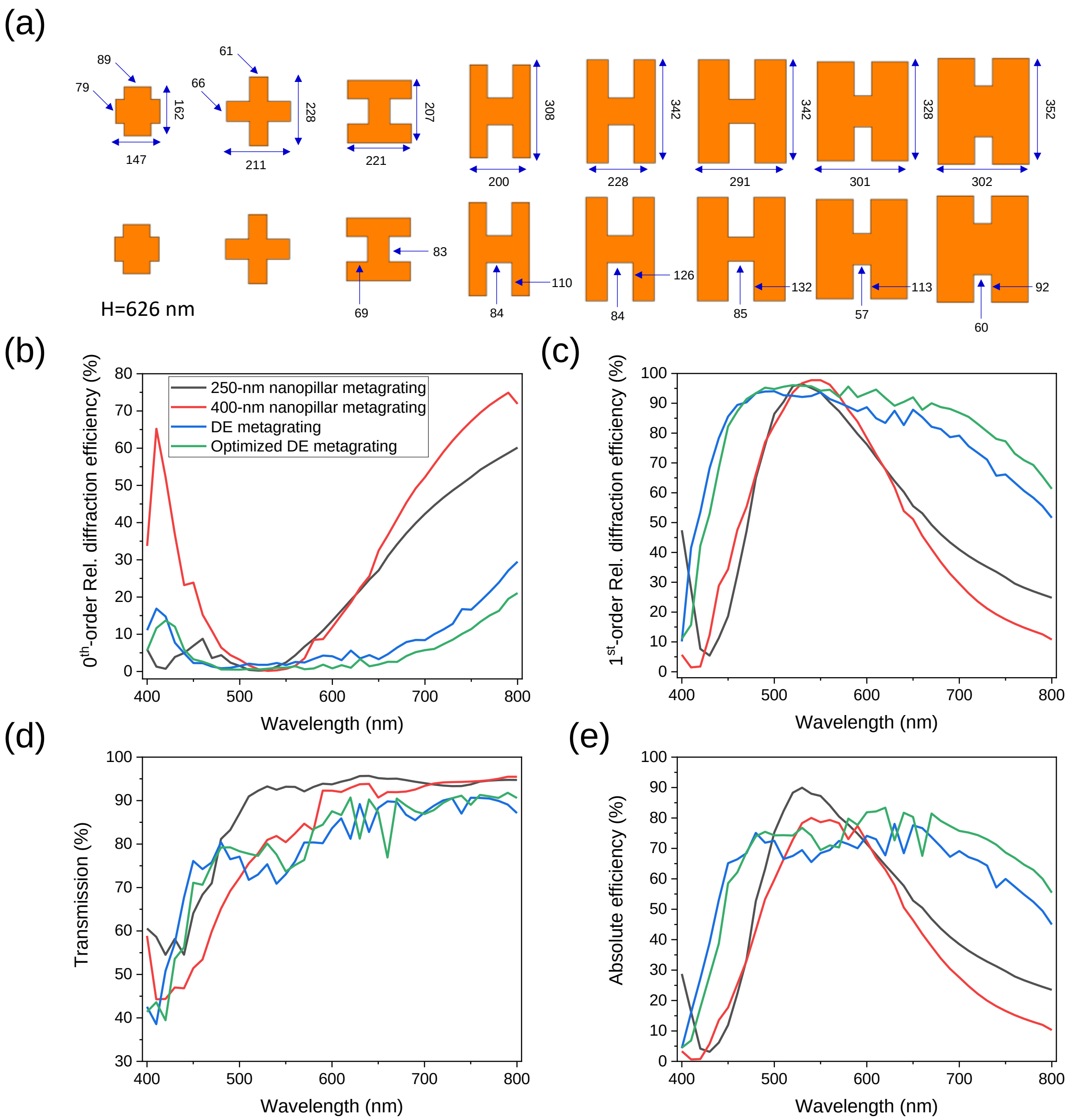

Figure S14: Simulated efficiency comparison of metagratings and the layout of an optimized metagrating. (a) Layout of a particle swarm optimized and dispersion-engineered metagrating. The height of these nanostructures is 626 nm. (b) and (c) shows the powers of 0th and 1st diffraction orders divided by the power of transmitted light, respectively. The red and black curves correspond to nanopillar metagratings comprising nanopillars with 400-nm and 250-nm center-to-center distances. The blue curve corresponds to the dispersion-engineered (DE) metagrating shown in Fig. 1b. The green curves correspond to the metagrating shown in (a), which has the best performance. The 0th and 1st order relative diffraction efficiencies in case of the 250-nm nanopillar are low at blue wavelengths because of diffraction to high orders. (d) and (e) Transmission and diffraction efficiency plots for the four different metagratings. The efficiencies and transmission are averaged under x- and y-polarized incident light.

Reference:

[S1] R. C. Devlin, M. Khorasaninejad, W. T. Chen, J. Oh, and F. Capasso, "Broadband high-efficiency dielectric metasurfaces for the visible spectrum," *Proc. Natl. Acad. Sci. U.S.A.* **113**, 10473-10478 (2016).
